# Embryonic Ethanol Exposure Affects Early- and Late-Added Cardiac Precursors and Produces Long-Lasting Heart Chamber Defects in Zebrafish

**DOI:** 10.3390/toxics5040035

**Published:** 2017-12-01

**Authors:** Swapnalee Sarmah, James A. Marrs

**Affiliations:** Department of Biology, Indiana University Purdue University Indianapolis, 723 West Michigan, Indianapolis, IN 46202, USA

**Keywords:** fetal alcohol spectrum disorder, congenital heart defects, cardiac defects in zebrafish, ethanol exposure

## Abstract

Drinking mothers expose their fetuses to ethanol, which produces birth defects: craniofacial defects, cognitive impairment, sensorimotor disabilities and organ deformities, collectively termed as fetal alcohol spectrum disorder (FASD). Various congenital heart defects (CHDs) are present in FASD patients, but the mechanisms of alcohol-induced cardiogenesis defects are not completely understood. This study utilized zebrafish embryos and older larvae to understand FASD-associated CHDs. Ethanol-induced cardiac chamber defects initiated during embryonic cardiogenesis persisted in later zebrafish life. In addition, myocardial damage was recognizable in the ventricle of the larvae that were exposed to ethanol during embryogenesis. Our studies of the pathogenesis revealed that ethanol exposure delayed differentiation of first and second heart fields and reduced the number of early- and late-added cardiomyocytes in the heart. Ethanol exposure also reduced the number of endocardial cells. Together, this study showed that ethanol-induced heart defects were present in late-stage zebrafish larvae. Reduced numbers of cardiomyocytes partly accounts for the ethanol-induced zebrafish heart defects.

## 1. Introduction

Prenatal alcohol exposure produces birth defects, including craniofacial defects, organ deformities, cognitive impairment, and sensorimotor disabilities, which are collectively termed as fetal alcohol spectrum disorder (FASD). FASD incidences are as high as 2–5% of children born in the US [1,2], and FASD prevalence is higher in lower socioeconomic populations [3,4]. Different forms of congenital heart defects (CHDs) are present in FASD patients [5]. CHDs are costly and life threatening global health problems, producing considerable morbidity and mortality. Greater than half of CHDs in the United States require at least one invasive surgery (www.childrensheartfoundation.org).

Heart development is regulated by molecular, cellular, and environmental factors. Mammalian heart develops from two types of cardiac progenitors, first heart field (FHF) and second heart field (SHF) cells [6]. These two cardiac fields can be distinguished by their spatiotemporal differentiation patterns and their ultimate contributions to the heart [7,8]. FHF differentiates initially at the lateral plate mesoderm, producing the linear heart tube [9]. The SHF differentiates next, and SHF precursors incorporate in both poles of the heart tube, elongating the heart tube [7,10,11]. Ultimately, the left ventricle is formed from the FHF cells. The right ventricle, outflow tract, parts of atria, and inflow tract are derived from the SHF cells [8]. The third key cardiac progenitor population is the cardiac neural crest, which invade the nascent heart and contribute cells to the outflow tract [6]. Cardiac neural crest is essential for the outflow tract remodeling [6]. Interfering with any of these progenitor populations leads to CHDs [9]. 

In zebrafish, specified cardiac progenitors migrate during gastrulation to reach the posterior half of the anterior lateral plate mesoderm at 12 hours post fertilization (hpf) [9,12]. These progenitors start to express cardiac specific gene *nkx2.5* [13,14]. FHF myocardial precursors fuse to form the heart cone around 19 hpf [15], which can be visualized by *myl7* expression [12]. At that time, *islet1* expressing SHF progenitor cells are found close to the heart cone [10]. At 24 hpf, differentiated FHF derived cardiomyocytes organize to form the linear heart tube [12]. Islet1 positive SHF cells are located at the outflow pole, anterior to the linear heart tube at that stage, which are recruited into the heart later (~24–38 hpf) and generate the distal part of the ventricle [7,10,11,16]. The zebrafish cardiac neural crest migrates and incorporates into the heart in two waves, contributing cells to different parts of heart at different developmental times [17,18,19]. Cardiac neural crest derived cardiomyocytes are mainly found at the base of the ventricle in zebrafish [19]. Like myocardium, endocardium is derived from the endocardial cells that are specified at the same time as the myocardial cells and migrate along with the cardiac progenitors [12]. Genetic or environmental effects produce inappropriate numbers of cardiomyocytes or endocardial cells, leading to CHDs [9,12]. 

Developmental responses to embryonic ethanol exposure are conserved between different vertebrates [20,21,22,23,24,25,26,27]. The zebrafish model is instrumental for lineage tracing because of the availability of transgenic animals, and embryos show outstanding optical clarity [7,11,28]. Short maturation period (approximately 3 months) allows cost effective evaluation of embryonic ethanol exposure effects in adults [29]. Numerous zebrafish studies have unraveled genetic, molecular, and cellular mechanisms of cardiac development and function in these highly conserved cardiogenic pathways and cellular developmental frameworks [9,12,30,31]. Our previous work illustrates the utility of the zebrafish for studying heart development mechanisms disrupted by embryonic ethanol exposure, showing that ethanol exposure disrupted cardiogenic gene expression and delayed myocardial cone formation [29,32]. Chronic ethanol-exposed (2–48 hpf) embryos had smaller, aberrantly looped heart or straight heart [32]. Instead of bean-shaped atrium and ventricle that are connected to each other with a narrow atrioventricular (AV) canal in control embryos, ethanol-treated embryos had misshapen atrium and ventricle that were oriented anterior-posteriorly [32]. In addition, those embryos had defective AV valves and deformed endocardium [29,32].

To establish zebrafish as a useful FASD model, it is important to know whether ethanol-exposed defects seen during early development persist at later stages of life. Our studies analyzing the effects of ethanol on AV valves showed that valve defects seen in embryos persisted in two month old zebrafish [29]. It is not known whether initially deformed zebrafish heart chambers remain defective, or they recover a normal-shaped heart over the course of time. To understand the FASD pathogenesis, experiments were designed to examine whether ethanol exposure differentially affects early differentiated and late differentiated cardiac fields. This study showed that ethanol-induced chamber deformities present at embryonic stages persisted in larval zebrafish. Interestingly, ventricles in these larvae had massive cardiac damage. Ethanol exposure delayed myocardial differentiation of FHF and SHF and reduced the numbers of early and late differentiated cardiac precursors and endocardial cells. 

## 2. Materials and Methods

### 2.1. Zebrafish Husbandry and Ethanol Treatment

Zebrafish (*Danio rerio*) were maintained under standard laboratory conditions [33] and following a protocol approved by Indiana University-Purdue University Indianapolis School of Science Institutional Animal Care and Use Committee (IACUC). *Tg*(*myl7*:*GFP*), *Tg*(*kdrl*:*EGFP*), *Tg*(*fli1*:*EGFP*), *Tg*(*myl7*:*nlsKikGR*) [11] transgenic lines were used in this work. *Tg*(*myl7*:*nlsKikGR*) fish contain KikGR fluorophore that fluoresces brightly with a green signal. A brief exposure to UV light permanently photoconverts KikGR from green fluorophore to a red fluorophore. *Tg*(*myl7*:*nlsKikGR*) embryos were exposed to UV light through DAPI filter to convert to red fluorophore.

Embryos that were maintained in embryo medium until 3 hpf were transferred to either 100 mM ethanol (E100) or 150 mM ethanol (E150) in embryo medium for ethanol treatment. At 24 hpf, ethanol solutions were replaced with fresh embryo medium. Untreated and ethanol treated larvae (severely malformed embryos were excluded) were housed in the fish system at 5 days post fertilization (dpf) and raised maintaining same conditions.

### 2.2. Histology

For histology analyses, 18 dpf larvae were fixed in 4% paraformaldehyde (PFA) in phosphate buffered saline (PBS), dehydrated to 95% ethanol, embedded in JB-4 resin (Polysciences, Warrington, PA, USA), sectioned at 5 μm thickness using a Leica RM2265 microtome. Sections were then stained with Masson’s trichrome dye. 

### 2.3. Immunofluorescence

Embryos were fixed in 4% paraformaldehyde and whole mount immunostaining was performed using primary antibody against Islet-1/2 (supernatant 1:50; Developmental Studies Hybridoma Bank, 39.4D5, Iowa City, IA, USA) and myosin heavy chain (supernatant 1:50; Developmental Studies Hybridoma Bank, MF 20). Alexa-Fluor 555-conjugated anti-mouse secondary antibody (1:500; Molecular Probes, Eugene, OR, USA) was used. 

### 2.4. Fluorescence in Situ Hybridization (FISH)

Whole mount FISH was performed using dig-labeled riboprobe to detect *nkx2.5*. Twenty four hour old embryos were fixed overnight in 4% PFA at 4 °C, washed in PBT (PBS containing 0.1% Tween 20), dehydrated to methanol, and stored at −20 °C. Embryos were permeabilized with proteinase K after rehydration, re-fixed in 4% PFA for 20 min at room temperature and washed. Embryos were then incubated in hybridization buffer for 2 h followed by incubation in hybridization buffer containing riboprobe overnight at 70 °C. Next day, following stepwise wash to 2× SSC, 0.05× SSC and PBT, the embryos were incubated in blocking solution (1× maleic acid buffer, 2% BSA and 2% normal goat serum) for 2 h at room temperature. The embryos were incubated in anti-Dig POD (1:1000; Roche, Mannheim, Germany) in blocking solution overnight at 4 °C. Finally, the embryos were washed with PBS and developed with tyramide signal amplification kit (T20912, Molecular Probes, Eugene, OR, USA). Alexa fluor-488 labeled tyramide (1:100) with hydrogen peroxide was added to the embryos for 60–90 min at room temperature [34]. The embryos were washed three times for 10 min with PBT, and then, immunostaining protocol described above was followed using MF 20 antibody (1:50) (Developmental Studies Hybridoma Bank, Iowa City, IA, USA) to detect myosin heavy chain. Alexa-Fluor 555-conjugated goat-anti mouse (1:200) secondary antibody (Molecular Probes, Eugene, OR, USA) was used. Embryos were imaged using a Zeiss LSM 700 confocal microscope (Carl Zeiss Microscopy, Thornwood, NY, USA) [34].

### 2.5. Microscopy and Cell Counting

Leica MZ12 dissecting stereomicroscope acquired with a color Leica DFC290 camera (Leica Microsystems, Inc., Deerfield, IL, USA) was used to acquire bright field images. Zeiss LSM 700 (Carl Zeiss Microscopy, Thornwood, NY, USA) confocal microscope was used to acquire confocal images. 3D renderings of the confocal images were produced from image volumes using either Volocity (PerkinElmer, Waltham, MA, USA) or ZEN software (2.1, Release Version 11.0, Carl Zeiss Microscopy, Jena, Germany, 1997–2015). Islet-1 labelled cells, *myl7*:*nlsKikGR* positive cells or *fli1*:*EGFP* positive cells were counted in 3D images using either Volocity or ZEN software.

### 2.6. Statistical Analysis

One-way ANOVA with post hoc Tukey HSD analyses were done to compare different groups of embryos (http://astatsa.com/OneWay_Anova_with_TukeyHSD/). 

## 3. Results

### 3.1. Ethanol-induced Cardiac Chamber Defects Occurred during Zebrafish Embryonic Development and Persisted in Larvae

**Figure 1:**
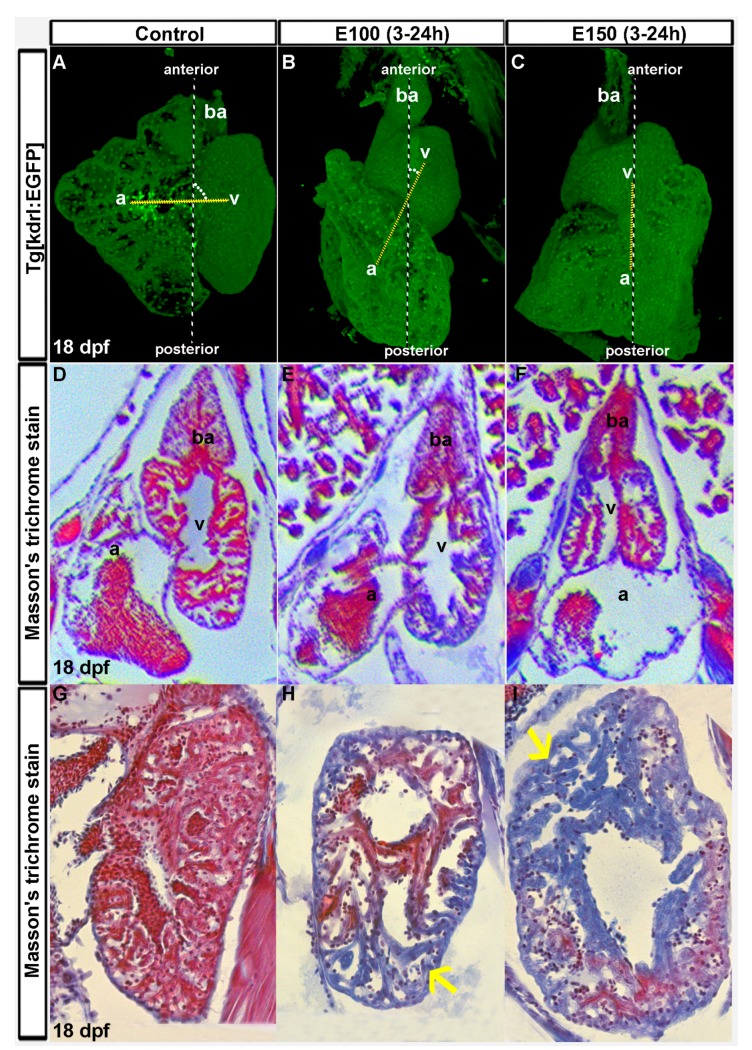
Ethanol-induced cardiac chamber defects occurring during embryogenesis persisted in older zebrafish larvae. (**A**–**C**) 3D renderings of confocal sections of *Tg*(*kdrl*:*EGFP*) show closely attached atrium and ventricle residing side by side in the control larva (**A**); ventricles are on the top of the atria in ethanol-exposed larvae (**B**,**C**); Dotted yellow line represents the line through AV valves connecting atrium and ventricle. The angle between yellow line and antero-posterior axis is shown by white dots; (**D**–**F**) Masson’s trichrome stained histology sections at the atrioventricular canal region showing both atrium and ventricle revealed defective anatomy of the ethanol-exposed larvae (**E**,**F**) compared to control (**D**); (**G**–**I**) Masson’s trichrome stained histology sections of the ventricle showed healthy looking cardiac muscle (red colored trabeculae) in control larva (**G**); and myocardial damage (blue colored trabeculae, yellow arrows) in ethanol-exposed larvae (**H**,**I**).

Zebrafish embryos were exposed to ethanol (E100 or E150) from 3 to 24 hpf and raised in normal conditions afterward. To investigate the long lasting effects of embryonic ethanol exposure on zebrafish heart, larvae were analyzed at day 18. Transgenic *Tg*(*kdrl*:*EGFP*) fish were used to visualize the heart. A total of eighteen control, nineteen E100, and seventeen E150 larvae from three different experiments were examined. Three-dimensional reconstruction of confocal images from *Tg*(*kdrl*:*EGFP*) of the heart of control larvae showed closely-associated atrium and ventricle residing side by side (Figure 1A). Larvae that were exposed to ethanol during embryogenesis had defective but variable heart anatomy. Almost all ethanol-exposed larvae had smaller ventricles than the controls. But the relative positions of atria and ventricles in those larvae varied with ventricles localized anterior to the atrium. An imaginary line through AV valves connecting atrium and ventricle and the antero-posterior body axis produces 90° angle in control larvae, but acute angles in ethanol-exposed larvae. Severely defective larvae had ventricles sitting on the top of the atria producing zero angles (Figure 1B,C). Similar variations in the orientation of the chambers were seen in ethanol-exposed embryos at 50 hpf, which was reported previously [32]. 

To extend the confocal microscopy data, 18 dpf larvae hearts were sectioned and stained with Masson’s trichrome, which stains muscle fibers red and collagenous fibrous tissue blue [35]. Low magnification images of the heart sections at the atrioventricular canal (AVC) region showed defective anatomy in atria and ventricles of the ethanol-exposed larvae hearts. Surprisingly, histology sections from a few ethanol-exposed larvae displayed blue coloration in massive trabeculae (irregular muscular columns projecting from the inner surface of the ventricle) areas (Figure 1D–F). Nine control larvae were examined and all trabeculae in control hearts stained intense red indicating healthy cardiac muscle. Four out of nine E100 and four out of eight E150 larvae showed blue coloration in the trabeculae muscle fibers reporting collagenous fibrotic tissues. This result indicated significant cardiac fibrosis in ethanol-exposed larvae possibly due to cardiac damage (Figure 1G–I). Thus, we conclude that exposure to ethanol during early development causes heart deformity and cardiomyopathy in zebrafish.

### 3.2. Embryonic Ethanol Exposure Reduced Early and Late Added Cardiomyocytes in the Heart

**Figure 2:**
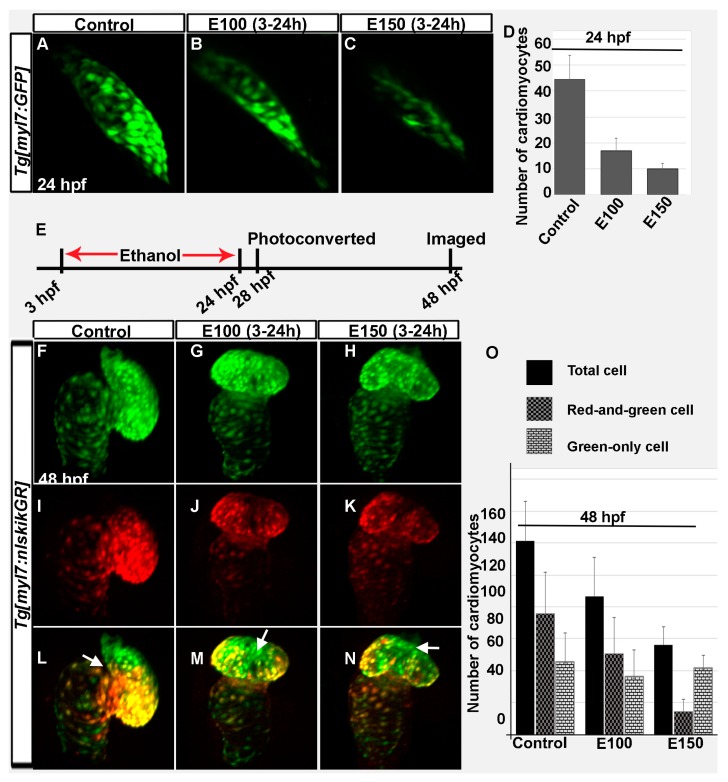
Ethanol exposure during embryogenesis reduced the number of early- and late-added cardiomyocytes in the heart. (**A**–**C**) 3D renderings of confocal sections of *Tg*(*myl7*:*GFP*) embryos showed GFP positive FHF derived cardiomyocytes in the linear heart tube in the control (**A**) and ethanol-exposed embryos (**B**,**C**); (**D**) Graph shows reduced number of FHF derived cardiomyocytes in ethanol-exposed embryos at 24 hpf. *p* < 0.001; (**E**) Schematic shows the time of ethanol treatment, photoconversion, and image acquisition in this experiment; (**F**–**N**) 3D renderings of confocal sections of photoconverted *Tg*(*myl7*:*nlskikGR*) embryos showed hearts in the control and ethanol-treated embryos. Note the green-only cardiomyocytes (cardiomyocytes added after photoconversion) in the anterior region of the ventricle in control embryos (**A**,**L**); but in the mid-ventricular region in ethanol-treated embryos (**G**–**N**); White arrows: green-only cells; (**O**) Graph shows the quantification of total, red-and-green (early-added cardiomyocytes) and green-only (late-added cardiomyocytes) cardiomyocytes at 48 hpf.

Previous experiments showed that embryos exposed to ethanol from 2 to 48 hpf had smaller, misshapen hearts (occasionally straight heart) when analyzed at 52 hpf [32]. Ethanol-treated embryos had reduced numbers of ventricular cardiomyocytes [32]. The treatment period of that experiment (2–48 hpf) comprised all critical heart developmental stages of zebrafish [12]. In this study, embryos were treated in ethanol from 3 to 24 hpf and analyzed at 48 hpf. This protocol examined a shorter exposure period, ending when the FHF cardiomyocytes organize into a functional heart tube (24 hpf). The hearts in ethanol-exposed embryos were smaller compared to control embryos. Confocal imaging and cell counting of *Tg*(*myl7*:*GFP*) embryos showed significantly reduced number of cardiomyocytes in the linear heart of ethanol-exposed embryos at 24 hpf (Figure 2A–D). Control embryos had 44.43 ± 9.36 cells, whereas, E100 had 16.78 ± 4.87 (*p* < 0.001), and E150 had 9.8 ± 2.14 cells (*p* < 0.01). Next, the effects of ethanol on early and late incorporated cardiomyocytes in the heart were examined using *Tg*(*myl7*:*nlsKikGR*) embryos, which labels cardiomyocytes with a green-to-red photoconvertible protein KikGR. *Tg*(*myl7*:*nlsKikGR*) green-to-red photoconvertible protein KikGR fluoresces green before activation by a brief exposure to UV light, which photoconverts KikGR to a red fluorophore. *Tg*(*myl7*:*nlsKikGR*) embryos were photoconverted at 28 hpf and imaged at 48 hpf. The cardiomyocytes imaged at 48 hpf that were photocoverted at 28 hpf (primarily FHF origin) showed both red and green, but the cardiomyocytes incorporated after photoconversion (SHF and cardiac neural crest origin) showed only green fluorescence. Counting of red-and-green and green-only cardiomyocytes showed reduction of both types of cells in E100 embryos compared to control (Control: red-and-green, 75.6 ± 26.3 and green-only, 45.6 ± 17.8; E100: red-and-green, 50.5 ± 22.7 and green-only, 36.1 ± 16.9). E150 embryos had significantly reduced red-and-green cells (14.5 ± 7.8) than green-only cells (41.8 ± 7.9) (Figure 2F–O). Total numbers of cardiomyocytes were significantly reduced after ethanol exposure (Control: 121.1 ± 24.7; E100: 86.62 ± 26.3; E150: 56.3 ± 17.83; control vs. E100, *p* < 0.05; control vs. E150, *p* < 0.01) (Figure 2O). Moreover, cardiomyocytes added later (green-only) in the control embryos were mostly distributed in the ventricle near the outflow track and at the base of the ventricle (Figure 2F–L). However, ethanol-treated embryos often had late-added cardiomyocytes in the mid-region of the ventricular wall (Figure 2G–N). Taken together, ethanol exposure reduces both early- and late-added cardiomyocytes and may change the distribution of late-added cardiomyocytes.

### 3.3. Early Ethanol Exposure Delayed Cardiac Differentiation

**Figure 3:**
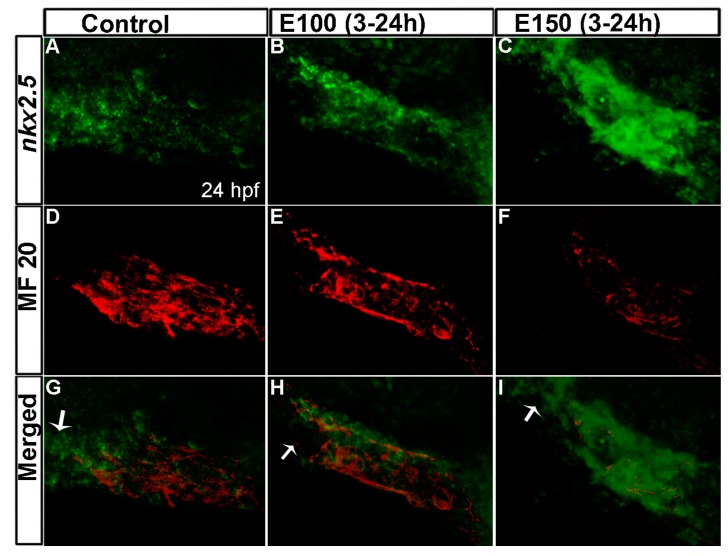
Myocardial differentiation was delayed in ethanol-exposed embryos. (**A**–**C**) In situ hybridization detecting *nkx2.5* expression showed weak *nkx2.5* expression in the linear heart tube (indicated fewer undifferentiated FHF derived cells) but strong expression at the outflow pole (indicated arrival of second heart field (SHF) progenitors) in the control embryo (**A**); E100 embryos displayed strong *nkx2.5* expression in the linear heart tube but no expression at the outflow pole (**B**); E150 embryos showed strong *nkx2.5* expression in the linear heart tube and at the outflow pole (**C**); (**D**–**F**) Strong MF20 antibody staining in the linear heart tube in control embryos labeled differentiated cardiomyocytes (**D**); MF 20 staining was weaker in the heart tube in ethanol-treated embryos (**E**,**F**); (**G**–**I**) Co-labeled images showed myocardial differentiation delay in ethanol-exposed embryos. White arrows: pointing to the outflow pole.

Ethanol exposure during zebrafish embryogenesis perturbed expression of cardiac transcription factors [32]. To understand the underlying cause of reduced cardiomyocyte numbers, cell proliferation and cell death were analyzed in the cardiac cone at 20 hpf and in the linear heart tube at 24 hpf. There was no change in proliferation or cell death after ethanol exposure at the stages tested (data not shown). To analyze cardiac differentiation, embryos were co-labeled to detect myosin II heavy chain (a differentiated cardiomyocyte marker) by MF 20 antibody immunofluorescence, and *nkx2.5* (a cardiac progenitor cell marker) by whole mount fluorescence in situ hybridization [9,12]. This showed weak expression of *nkx2.5* and strong MF 20 signal in the linear heart tube in control embryos, which indicates normal cardiac progenitor differentiation into cardiomyocytes (Figure 3A,D). The expression of *nkx2.5* was stronger but MF 20 staining was weaker in the heart tubes of ethanol-exposed embryos, suggesting that there were more cells in the progenitor state than the differentiated state (Figure 3B–I). The difference was more pronounced in E150 embryos than in E100 (Figure 3B–I). This result suggested that ethanol exposure delayed myocardial differentiation.

Control embryos showed strong expression of *nkx2.5* at the outflow pole that was devoid of MF 20 staining (Figure 3G). SHF progenitors reach the outflow pole region of heart tube at 24 hpf and start to express *nkx2.5* [7]. This was observed in our control embryos (Figure 3A,D,G), but E100 embryos displayed no *nkx2.5* expression at the outflow pole (Figure 3B,E,H), possibly because of the delay in SHF progenitor’s arrival. E150 showed strong *nkx2.5* expression in all parts of the heat tube, including the anterior part of the heart tube (Figure 3C). This may be due to labeling of undifferentiated FHF progenitors that were still migrating to form the heart tube.

**Figure 4:**
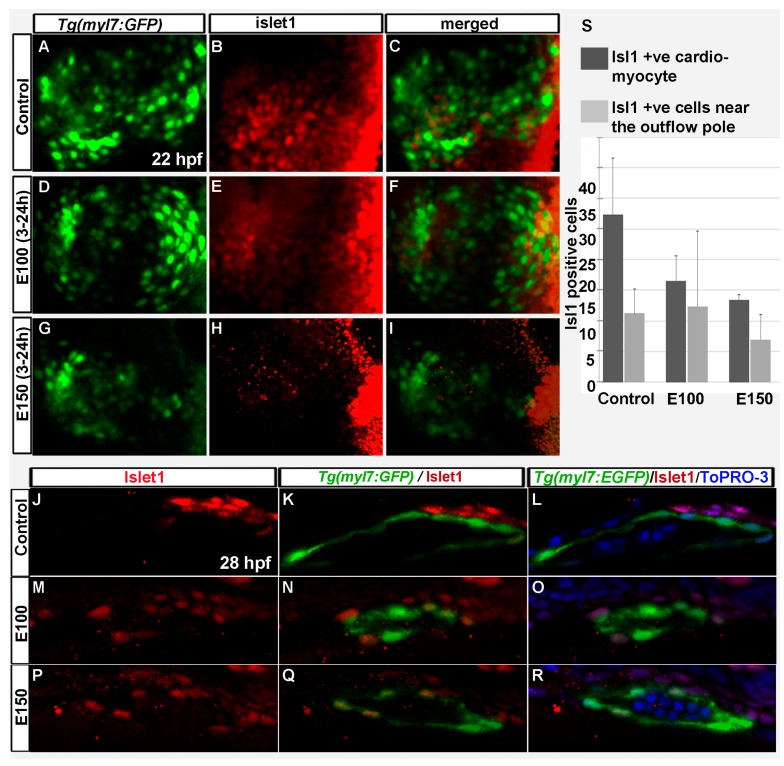
Embryonic ethanol exposure reduced second heart field precursors. (**A**–**I**) Anti-Islet1 antibody stained *Tg*(*myl7*:*GFP*) embryos showed Islet1 positive second heart field precursors in the rotating heart cone in control embryos (**A**–**C**) at 22 hpf; ethanol exposed embryos showed reduced number of Islet1 positive cells in the heart cones (**D**–**I**) at 22 hpf; (**J**–**R**) Anti-Islet1 antibody stained *Tg*(*myl7*:*GFP*) embryos showed second heart field derived cardiomyoctes in the heart (Myl7 and Islet1 double positive; red and green) and second heart field precursors (Islet1 positive, red) near the out flow pole in the control embryos (**J**–**L**) and ethanol-exposed embryos (**M**–**R**) at 28 hpf; (**S**) Graph shows the quantification of the Islet1/Myl7 double positive cardiomyocytes in the heart tube and Islet1 positive cells near the outflow pole at 28 hpf.

Islet-1 is a critical regulator of SHF development, and SHF progenitors express Islet-1 [10,36]. To test how ethanol exposure affected SHF progenitors, antibody staining was performed in *Tg*(*myl7*:*GFP*) embryos to label Islet-1 at 22 and 26 hpf. Confocal imaging was done, and Islet-1 expressing cells were counted. Control embryos showed Islet-1 positive cells intermingled with *myl7*:*GFP* positive cells in the rotating heart cone at 22 hpf (Figure 4A–C). E100 embryos had fewer Islet-1 positive cells in the heart cone (Figure 4D–F). E150 embryos had no Islet-1 positive cells at 22 hpf in the heart cone (Figure 4G–I). At 26 hpf, Islet-1 positive cells were found in the heart and expressing *myl7*:*GFP*. Some Islet-1 cells were located in the outflow pole, close to the ventricle (Figure 4J–R). Similar distributions were observed in the control and ethanol treated embryos (Figure 4J–R). However, the numbers of cardiomyocytes expressing both *myl7*:*GFP* and Islet-1 were significantly reduced in ethanol-exposed embryos (Control: 27.4 ± 9.0, E100: 16.5 ± 4.0, E150: 13.5 ± 0.7; Control vs. E100 *p* < 0.05, Control vs. E150 *p* < 0.01) (Figure 4S).

### 3.4. Embryonic Ethanol Exposure Reduced Endocardial Cell Number

**Figure 5:**
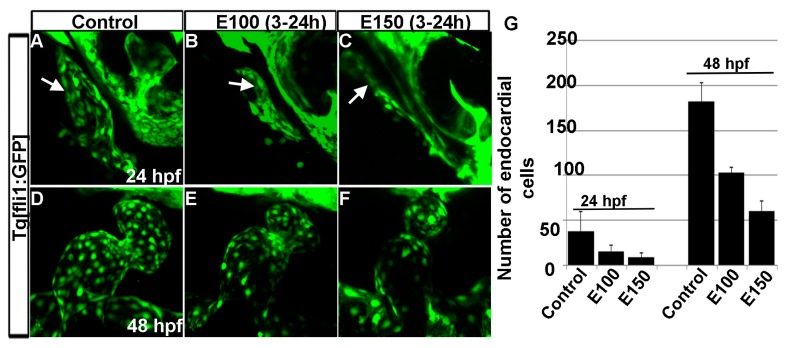
Ethanol exposure reduced endocardial cell numbers. (**A**–**C**) *Tg*(*fli1*:*EGFP*) embryos showed endocardial lining in the linear heart tube in control embryo (**A**) and in ethanol-exposed embryos (**B**,**C**) at 24 hpf; (**D**–**F**) *Tg*(*fli1*:*EGFP*) embryos showed normal-shaped endocardium in the control embryo (**D**) and deformed endocardium with fewer endocardial cells in ethanol-exposed embryos at 48 hpf (**E**,**F**); (**G**) Graph shows the quantification of the endocardial cells at 24 and 48 hpf. White arrow: endocardial lining.

Our previous studies showed that ethanol exposure changed the shape of the endocardium [18]. To understand the endocardium defects, endocardial cells were counted using *Tg*(*fli1*:*EGFP*) embryos, which labels endothelial and endocardial cells. Counting GFP positive cells in three dimensional *Tg*(*fli1*:*EGFP*) images showed a significant reduction in the numbers of endocardial cells after ethanol exposure (Figure 5A–G). At 24 hpf, control embryos had 44.43 ± 15.72 cells, whereas, E100 had 15.75 ± 6.36 (*p* < 0.01), and E150 had 9.0 ± 5.1 cells (*p* < 0.001). At 50 hpf, endocardial cell number increased in the control embryos to 182 ± 20.79. E100 embryos had 103 ± 5.95 (*p* < 0.05), and E150 had 60 ± 11.2 (*p* < 0.001) cells at that stage (Figure 5G). This result showed that ethanol affected endocardium development.

## 4. Discussion

Although cardiovascular disease is a leading cause of death worldwide [37], the etiology of CHDs are mostly unknown. In addition to various chemical compounds ubiquitously present in the environment, exposure to illicit drug and alcohol during prenatal development, and maternal nutritional deficiencies are linked to CHDs [38,39]. Animal studies showed that ethanol exposure during embryonic development led to heart development defects [29,32,40,41,42]. Zebrafish studies showed that ethanol exposure causes cardiogenesis defects leading to abnormal structure of chambers and atrtioventricular canal in embryos [29,32]. Our previous work examining AV valves in late-stage larvae (18 and 35 day old) and juvenile (2 month old) zebrafish exposed to ethanol during embryogenesis revealed faulty AV valves in those fish. Ethanol-exposed fish had smaller valve leaflets. Moreover, the shape of those leaflets were different and did not allow complete closure of the AV junctions [29]. It was not known whether the chamber defects seen during early development persisted in older animals. This study demonstrated defective heart chambers in larvae exposed to ethanol during embryogenesis at day 18 in addition to the deformed valves, which suggests that the defects occurring during early development persisted at later stages in zebrafish. Surprisingly, histological analyses of those hearts showed cardiac fibrosis in the cardiac muscle tissues. Ethanol exposed larvae might suffer from cardiac injury leading to fibrosis. To our knowledge, this is the first report that links cardiac injury phenotype in animals prenatally exposed to ethanol. Attention should be given to determine potential connections between prenatal alcohol exposure and cardiomyopathy. Properly designed rigorous epidemiologic study should be carried out to better understand the association of maternal alcohol consumption with risk of heart defects and diseases. Zebrafish has tremendous capacity to regenerate its heart after injury [43]. It will be interesting to study how the fish that were initially exposed to ethanol repair damaged cardiac muscle. 

Our previous studies showed that chronic ethanol exposure from 2 to 48 hpf reduced the size of the ventricle in zebrafish embryos [32]. Reduced ventricle size was correlated with reduced cardiomyocyte number and smaller cardiomyocyte surface area [32]. The current study demonstrated that even if the embryos were exposed to ethanol from 3 to 24 hpf, the period when the linear heart tube formed from FHF cells, cardiomyocyte numbers were significantly reduced in the ventricle. The KikGR photo conversion assay indicated that early- and late-added cardiomyocyte populations were affected by ethanol exposure. However, the result is not very conclusive. The fact that ethanol exposure delayed cardiomyocyte differentiation, as evident by the *nkx2.5* and MF 20 staining, made it hard to conclude that the green-only cells detected at 48 hpf were exclusively the cells added later. There is a likelihood that a green-only cells could also be a FHF derived cells present in the linear heart that was in an undifferentiated precursor state at the time of photoconversion and did not have *myl7* expression. Those cells might express *myl7* and hence *nlsKikGR* after the photo conversion was done. This idea explains why more green-only cells were detected in E150 embryos than E100 at 48 hpf. Although ANOVA analyses comparing all groups of embryos showed significantly reduced total number of cardiomyocytes in ethanol-exposed embryos, either red-and-green only cells or green-only cells were not significantly different in ethanol-exposed embryos comparing to control (except red-and-green only cells in E150, Control vs. E150 *p* < 0.01). Future experiments analyzing cell lineages will provide definitive data. Experiments examining Islet-1 expression during heart cone formation showed reduced SHF precursors after ethanol exposure. Islet-1 positive cardiomyocytes were significantly reduced in the heart tube in ethanol-exposed embryos.

Previously, our studies showed that ethanol exposure (2–48 hpf) produces misshapen endocardium [32]. Ethanol exposure from 3 to 24 hpf induced similar endocardial defects. Endocardium develops alongside the myocardium, and defective myocardium shape can indirectly affect the shape of endocardium. The current study showed that ethanol exposure reduced the number of endocardial cells. Defective endocardium could arise independently of the myocardium defect or be an indirect effect of defective myocardium. 

In summary, this study showed that ethanol-induced heart defects seen in zebrafish embryos were present in late-stage zebrafish larvae. Our study demonstrated reduction of first and second heart field cardiac precursors after ethanol exposure, which might partly account for the defects.
